# Smoking is an independent risk factor for nosocomial sinusitis in neurocritical patients: evidence from a retrospective cohort study

**DOI:** 10.3389/fneur.2025.1575578

**Published:** 2025-07-14

**Authors:** Qiong Chen, Qindi Zhang, Dongmei Wang, Man Yang, Linyan Lai, Daihong Cheng, Ling He, Xiaoyan Liao

**Affiliations:** ^1^Department of Neurology, Nanfang Hospital, Southern Medical University, Guangzhou, China; ^2^Nursing School, Southern Medical University, Guangzhou, China; ^3^Department of Neurology, Zengcheng Campus, Nanfang Hospital, Southern Medical University, Guangzhou, China; ^4^School of Nursing, Guangzhou Medical University, Guangzhou, China

**Keywords:** critical care, nasogastric intubation, neurology, nosocomial sinusitis, smoking

## Abstract

**Objectives:**

To determine the risk factors associated with nosocomial sinusitis in general neurology ward patients with nasogastric tube (NGT) intubation and neurocritical care unit (NCU) patients.

**Methods:**

A retrospective cohort study was conducted in a tertiary hospital, Guangzhou, China. We enrolled two groups of patients: (1) general neurology ward patients with NGT intubation between July 2018 and March 2021, and (2) those admitted to NCU between January 2021 and December 2022. Eligible patients had at least two head MRI/CT scans with no baseline sinusitis. Nosocomial sinusitis was determined by senior physicians through imaging. Demographic and clinical data were collected from electronic medical records. Multivariate logistic regression was employed to analyze risk factors.

**Results:**

In total, 206 patients were included. Of these, 75 were from general neurology wards and 131 were from NCU. Nosocomial sinusitis occurred in 58.7% (44/75) of general neurology patients with NGT intubation and 59.5% (78/131) of NCU patients. Prior intubation of either ETT or NGT (odds ratio [OR] = 2.60, 95% Confidence Interval [CI] 1.15–5.88), prior intubation of both ETT and NGT (OR = 6.17, 95%CI 1.82–20.94), being a current smoker (OR = 2.53, 95%CI 1.29–4.96), and prolonged NCU stays (OR = 1.05, 95%CI 1.01–1.09) were risk factors for nosocomial sinusitis in the total samples. Specifically, prior intubation of both ETT and NGT (OR = 2.31, 95%CI 1.42–34.15), being a current smoker (OR = 3.47, 95%CI 1.45–8.29), and prolonged hospital stay (OR = 1.05, 95%CI 1.02–1.10) were risk factors for nosocomial sinusitis in NCU patients.

**Conclusion:**

Nosocomial sinusitis was prevalent among both general neurology ward patients with NGT intubation and NCU patients. Strategies, such as routine screening, promoting smoking cessation, and alternative enteral feeding methods, may help reduce the incidence of nosocomial sinusitis in this population.

## 1 Introduction

Nosocomial sinusitis is prevalent but often overlooked in intensive care units (1). First documented in 1974 among four critically ill patients with nasotracheal intubation exhibiting facial pain, fever, and purulent nasal secretions (2), nosocomial sinusitis has since been identified in later studies as a sole cause in 16.2% of cases with unknown origin fevers in orotracheally intubated critically ill patients, and contributing factor in an additional 13.8% (3). The clinical significance of this condition extends beyond localized infection, as it may contribute to secondary complications such as bacteremia, sepsis, and ventilator-associated pneumonia (4), which are linked to prolonged hospital stays and increased mortality rates (5). In contrast, early detection and proactive management of nosocomial sinusitis in critically ill patients have resulted in reduced incidence of ventilator-associated pneumonia (6).

Reported incidence of nosocomial sinusitis in critically ill patients vary from 11.4 to 75%, depending on the study population and diagnostic methodology (7–9). Factors such as mechanical ventilation, prior nasotracheal and/or nasogastric intubation, antibiotic use, sedation, and a Glasgow Coma Scale (GCS) score of 7 or below have been associated with increased risk of nosocomial sinusitis (7, 9, 10). The evolution of neurocritical care as a distinct subspecialty since the 1980s has led to the establishment of Neurocritical Care Units (NCUs), dedicated to managing patients with severe neurological and neurosurgical conditions or systemic diseases with severe neurological symptoms (11). Compared to patients in general intensive care units, those cared for in NCUs experience reduced use of mechanical ventilation, intravenous sedation, and hemodynamic monitoring, but have longer hospital stays and higher rates of tracheostomy and need for nutritional support (12). These distinctive management paradigms suggest potentially divergent nosocomial sinusitis risk profiles in NCU patients that warrant specific investigation.

Current understanding of nosocomial sinusitis in neurocritical patients remains limited, with existing evidence primarily highlighting endotracheal tube (ETT) and/or nasogastric tube (NGT) intubation as a significant risk factor (9). Notably, radiographic sinusitis precedes pneumonia in 80.2% of NCU patients (13), reinforcing its potential role as a trigger for secondary infections. While the association of NGT with nosocomial sinusitis has been established (10), other determinants remain understudied. For instance, it remains unclear if smoking, a known risk factor for both stroke (14) and a dose-dependent risk factor for chronic sinusitis (15), plays a role in the development of nosocomial sinusitis in neurocritical patients. Furthermore, previous studies have predominantly focused on nosocomial sinusitis in intensive care patients, with limited attention to general neurology ward patients with NG intubation. It remains uncertain whether the impact of NGT intubation on nosocomial sinusitis incidence differs between NCU patients and those in general neurology wards.

This retrospective cohort study aimed to examine the incidence and risk factors for nosocomial sinusitis in NCU patients compared to general neurology ward patients requiring NGT intubation.

## 2 Methods

### 2.1 Study design and setting

This was a retrospective cohort study conducted at the neurology department of a 3,000-bed tertiary hospital in (blinded). The neurology department encompasses both a NCU and general neurology wards.

### 2.2 Participants

We enrolled two groups of patients: (1) patients who had undergone NGT intubation in general neurology wards between July 2018 and March 2021; and (2) patients admitted to the NCU between January 2021 and December 2022. Patients were identified through electronic medical records.

The inclusion criteria for patients in general neurology wards were (1) underwent NGT intubation, and (2) had at least two head imaging scans (either computed tomography [CT] or MRI) during consecutive hospital stays. The exclusion criteria were as follows: (1) patients diagnosed with sinusitis at the initial head imaging, (2) had a hospital stay of less than 48 h, or (3) had an NCU stay.

The inclusion criteria for NCU patients were as follows: (1) admitted to NCU during the study period, and (2) had at least two head imaging scans during consecutive hospital stay. The exclusion criteria for NCU patients were: (1) patients diagnosed with sinusitis at the initial head imaging, and (2) an NCU stay of ≤ 48 h.

An interval of 48–72 h after admission is widely accepted as the threshold for a nosocomial infection (16). Therefore, the primary outcome of this study was the occurrence of nosocomial sinusitis no less than 48 h after admission. Diagnoses were based on head CT or MRI, as assessed by senior radiologists of the Department of Medical Imaging. The presence of mucosal thickening or fluid in head images was indicative of sinusitis (1). Patients with scans suggestive of suspected tumor, fungus-like lesion, or other abnormality that did not correspond to simple sinusitis were excluded. The initial head scan performed within the first 48 h post-admission was designated as the baseline scan. Sinusitis diagnoses were limited to the time spent in either general neurology wards or NCU to maintain a standardized group of patients.

### 2.3 Data collection

A retrospective chart review was conducted. The dates and location of the insertion and removal of the ETT and/or NGT were recorded. We also documented ETT or NGT intubation (yes or no and duration), mechanical ventilation (yes or no), and antibiotic use (yes or no) prior to the diagnosis of sinusitis. Data for demographic information (i.e., age, sex, and smoking status), GCS score at admission, underlying medical conditions, length of NCU stay, length of hospital stay were collected from electronic medical records. Head images were reviewed. All data collection and analyses were performed without direct patient interaction. All participants underwent routine SARS-CoV-2 testing prior to admission and during their hospitalization. No instances of COVID-19 positivity were reported within our study cohort.

### 2.4 Statistical analysis

Descriptive statistics were displayed as frequencies and percentages for categorical variables, means and standard deviations for continuous variables, and medians with interquartile ranges for non-normal distributed quantitative variables. The comparison of categorical variables was performed using Pearson Chi-square or Linear-by-Linear Association, as appropriate. For continuous variables, the independent t-test or Mann–Whitney U test was applied.

For identifying risk factors, multivariant logistic regression was employed. Variables with a *p*-value under 0.1 in univariate analyses, which included sex, smoking status (current smoker, former smoker, or non-smoker), Prior ETT and/ or NGT intubation, Mechanical ventilation, Tracheostomy, presence of pneumonia, the length of NCU stay, and the length of hospital stay were included in the regression model. Individual who had not ceased smoking prior to their admission were classified as current smoker. Conversely, individual who had ceased smoking at least one months prior to their admission were classified as former smoker. Moreover, predictive factors identified by previous studies, such as age, antibiotics use, GSC scores were also included (7, 9). Odds ratios (ORs) with 95% confidence intervals (CIs) were calculated from the logistic regression model using likelihood ratio test.

Statistical analysis was conducted using SPSS version 25.0 (IBM Corp, Armonk, NY, United States). A *p* value of less than 0.05 was considered statistically significant.

### 2.5 Ethical approval

This study was approved by the Ethical Review Committee of Nanfang Hospital (NFEC-2024-037). Due to the retrospective and observational nature of the research, the review board exempted the requirement for informed consent. Authority to access patient data was granted by the designated statutory data custodian. To ensure privacy, all data were anonymized and secured with password protection.

## 3 Results

### 3.1 Demographical and clinical characteristics of the sample

**Figure 1:**
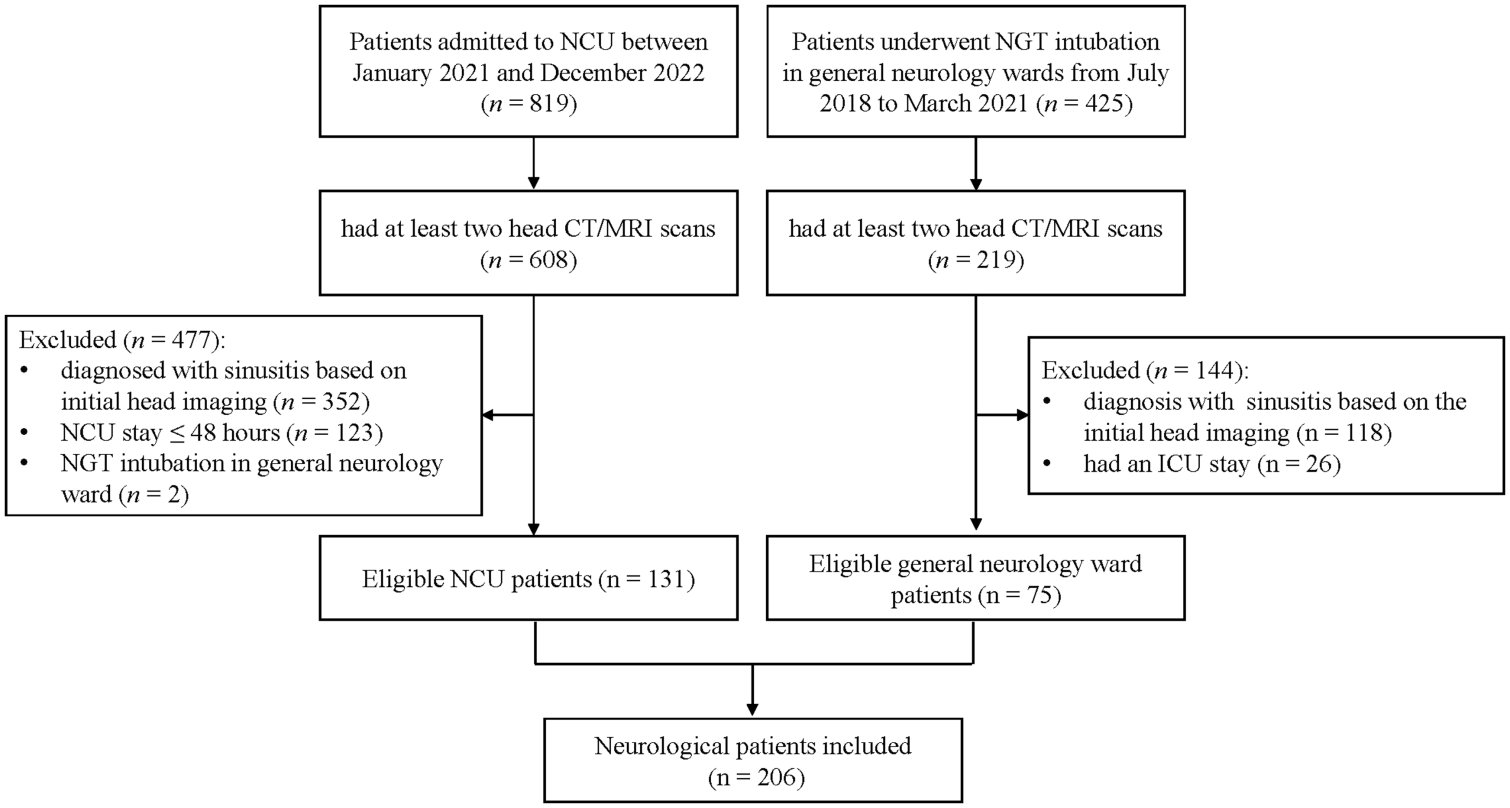
Flowchart of patient enrollment. CT, computed tomography; NCU, neurocritical care unit.

A total of 1,244 patients were screened, comprising 425 general neurology patients with NGT and 819 NCU patients. Of these, 827 met the inclusion criterion, including 219 general neurology patients and 608 NCU patients. One hundred and forty-four general neurology patients were excluded because of a diagnosis of sinusitis confirmed by initial head scans (*n* = 118) and having a NCU stay (*n* = 26). Four hundred and seventy-seven NCU patients were excluded because of a diagnosis of sinusitis confirmed by initial head scans (*n* = 352), NCU stay of less than 48 h (*n* = 123), and the location of the insertion of NGT being the general neurology ward (*n* = 2). The final cohort consisted of 206 patients. Of whom, 75 (36.4%) were from general neurology ward and 131 (63.6%) were from the NCU. The patient enrollment process is illustrated in Figure 1.

Of the 206 patients included, 132 (64.1%) patients were diagnosed with ischemic stroke, 41 (19.9%) were diagnosed with hemorrhagic stroke, and the remaining 33 (16.0%) were diagnosed with other conditions (e.g., encephalitis, seizures, or craniocerebral space-occupying lesion). Demographic and clinical characteristics of the study samples are shown in Table 1.

**Table 1:** Demographic and clinical characteristics of subjects (*n* = 206).

Variables	With NS (*n* = 122)	Without NS (*n* = 84)	*F/χ^2^/Z*	*P*
Setting, *n* (%)				
General neurology ward	44 (36.1)	31 (36.9)	0.015	0.902
NCU	78 (63.9)	53 (63.1)		
Male, *n* (%)	89 (73.0)	52 (61.9)	2.81	0.094
Age (years), mean ± SD	59.86 ± 16.92	60.80 ± 16.34	0.16	0.653
Smoking status, *n* (%)				
Never a smoker	55 (45.1)	52 (61.9)	9.04	0.011
Current smoker	54 (44.3)	20 (23.8)		
Former smoker	13 (10.7)	12 (14.3)		
Season, *n* (%)				
Spring	34 (27.9)	22 (26.2)	0.76	0.860
Summer	21 (17.2)	14 (16.7)		
Autumn	34 (27.9)	28 (33.3)		
Winter	33 (27.0)	20 (23.8)		
Diagnosis, *n* (%)				
Ischemic stroke	75 (61.5)	57 (67.9)	1.09	0.580
Hemorrhage stroke	27 (22.1)	14 (16.7)		
Others	20 (16.4)	13 (15.5)		
GCS score, mean ± SD	11.88 ± 3.33	12.17 ± 2.82	0.425	0.515
Intracranial intervention^#^, *n* (%)	58 (47.5)	38 (45.2)	0.11	0.745
Antibiotics use, *n* (%)	58 (47.5)	36 (42.9)	0.44	0.579
Sedative use, *n* (%)	28 (23.1)	6 (7.1)	9.02	0.003
Mechanical ventilation, *n* (%)	28 (23.1)	6 (7.1)	9.02	0.003
Tracheostomy, *n* (%)	7 (5.7)	1 (1.2)	2.74^§^	0.097
Prior ETT intubation^#^, *n* (%)	26 (21.3)	6 (7.1)	7.61	0.006
Prior NGT intubation^#^, *n* (%)	109 (89.3)	63 (75.0)	7.43	0.006
Prior ETT & NGT intubation^#^, *n* (%)	26 (21.3)	5 (6.0)	9.14^§^	0.003
Pneumonia, *n* (%)	52 (42.6)	21 (25.0)	6.75	0.009
NCU stays (days), Median (Q1, Q3)	5.0 (0, 11.0)	3.0 (0, 6.0)	−1.72	0.085
Hospital stays (days), Median (Q1, Q3)	16.0 (11.0, 22.0)	13.0 (9.0, 19.7)	−2.00	0.045

ETT, Endotracheal tube; GCS, Glasgow Coma Scale; NCU, neurocritical care unit; NGT, nasogastric tube; NS, nosocomial sinusitis; SD, Standard Deviation. Others included encephalitis, seizures, and craniocerebral space-occupying lesion. ^#^Prior to the diagnosis of nosocomial sinusitis. ^§^Linear-by-Linear Association.

### 3.2 Incidence of nosocomial sinusitis

Table 1 shows that nosocomial sinusitis was identified in 122 patients, which yielded an incidence rate of 59.2% (122/206). Nosocomial sinusitis occurred in 59.5% (78/131) of NCU patients and 58.7% (44/75) of general neurology patients with NGT. Specifically, 74.0% (97/131) of NCU patients underwent NGT intubation before their nosocomial sinusitis diagnosis. As shown in Table 2, among those who underwent NGT intubation, 67% (65/97) in the NCU and 58.7% (44/75) in the general neurology wards developed nosocomial sinusitis (χ^2^ = 1.27, *p* = 0.260).

**Table 2:** Comparisons between general neurology patients and NCU patients with NGT intubation.

Variables	NCU (*n* = 97)	General neurology (*n* = 75)	*F/χ^2^/Z*	*P*
Nosocomial sinusitis, *n* (%)	65 (67.0)	44 (58.7)	1.27	0.260
Male, *n* (%)	70 (72.2)	52 (69.3)	0.164	0.685
Age (years), mean ± SD	57.60 ± 15.87	65.16 ± 13.82	10.73	0.001
Smoking status, *n* (%)				
Never a smoker	49 (50.5)	39 (52.0)	10.85	0.004
Current smoker	42 (43.3)	20 (26.7)		
Former smoker	6 (6.2)	16 (21.3)		
Season, *n* (%)				
Spring	31 (32.0)	17 (22.7)	2.76	0.430
Summer	16 (16.5)	15 (20.0)		
Autumn	23 (23.7)	24 (32.0)		
Winter	27 (27.8)	19 (25.3)		
Diagnosis, *n* (%)				
Ischemic stroke	57 (58.8)	54 (72.0)	3.77	0.152
Hemorrhage stroke	25 (25.8)	11 (14.7)		
Others	15 (15.5)	10 (13.3)		
GCS score, mean ± SD	10.74 ± 3.27	13.37 ± 2.29	35.15	< 0.001
Intracranial Intervention, *n* (%)	57 (58.8)	14 (18.7)	28.05	< 0.001
Antibiotics use, *n* (%)	60 (61.9)	27 (36.0)	11.31	0.001
Sedative use, *n* (%)	32 (33.0)	1 (1.3)	27.34^§^	< 0.001
Mechanical ventilation, *n* (%)	32 (33.0)	1 (1.3)	27.34^§^	< 0.001
Tracheostomy, *n* (%)	8 (8.2)	0 (0.0)	6.45^§^	0.011
Prior NGT intubation^#^, *n* (%)	97 (100.0)	75 (100.0)		
Prior ETT intubation^#^, *n* (%)	30 (30.9)	1 (0.3)	24.93^§^	< 0.001
Prior ETT & NGT intubation^#^, *n* (%)	30 (30.9)	1 (0.3)	24.93^§^	< 0.001
Pneumonia, *n* (%)	58 (59.8)	7 (9.3)	46.81	< 0.001
NCU stays (days), Median (Q1, Q3)	15.0 (9, 20)	0.0 (0, 0)	−11.57	< 0.001
Hospital stays (days), Median (Q1, Q3)	16.0 (11.0, 24.5)	13.0 (9.0, 19.0)	−1.6	0.091

ETT, Endotracheal tube; GCS, Glasgow Coma Scale; NA, not applicable; NCU, neurocritical ca9re unit; NGT, nasogastric tube; SD, Standard Deviation. Others included encephalitis, seizures, and craniocerebral space-occupying lesion. ^#^Prior to the diagnosis of nosocomial sinusitis. ^§^Linear-by-Linear Association.

### 3.3 Risk factors for nosocomial sinusitis

Multivariate logistic regression analysis revealed that prior intubation of either ETT or NGT (OR = 2.60, 95%CI 1.15–5.88), prior intubation of both ETT and NGT (OR = 6.17, 95%CI 1.82–20.94), being a current smoker (OR = 2.53, 95%CI 1.29–4.96), and prolonged NCU stays (OR = 1.05, 95%CI 1.01–1.09) were risk factors for the incidence of nosocomial sinusitis in the total samples. Specifically, prior intubation of both ETT and NGT (OR = 2.31, 95%CI 1.42–34.15), being a current smoker (OR = 3.47, 95%CI 1.45–8.29), and prolonged hospital stays (OR = 1.05, 95%CI 1.02–1.10) were risk factors for the incidence of nosocomial sinusitis in NCU patients (Table 3).

**Table 3:** Risk factors for the occurrence of nosocomial sinusitis in the subjects.

Variables	Total (*n* = 206)			NCU (*n* = 131)		
	B	OR (95%CI)	*P*	B	OR (95%CI)	*P*
ETT or NGT intubation^a,#^	0.96	2.60 (1.15, 5.88)	0.022	0.67	1.95 (0.77, 4.95)	0.160
ETT and NGT intubation^a,#^	1.82	6.17 (1.82, 20.94)	0.004	2.19	2.31 (1.42, 34.15)	0.001
Smoking^&^			0.024			0.015
Current smoker	0.93	2.53 (1.29, 4.96)	0.007	1.24	3.47 (1.45, 8.29)	0.005
Former smoker	0.17	1.19 (0.48, 2.93)	0.708	−0.12	0.89 (0.19, 4.18)	0.882
Prolonged NCU stays	0.05	1.05 (1.01, 1.09)	0.029			
Prolonged hospital stays				0.05	1.05 (1.02, 1.10)	0.003

B, regression coefficients; CI, confidence interval; ETT, Endotracheal tube; NCU, neurocritical care unit; NGT, nasogastric tube; OR, odds ratio. ^a^Reference: neither ETT nor NGT intubation; ^&^Reference: non-smoker; ^#^Prior to the diagnosis of nosocomial sinusitis.

## 4 Discussion

In this retrospective cohort study, we observed a high incidence of nosocomial sinusitis in both general neurology ward patients with NGT intubation (58.7%) and NCU patients (59.5%). For NCU patients with NGT intubation, the incidence increased to 67%. After adjusting for age, sex, GCS score, and antibiotic use, we identified that NGT and/or ETT intubation, as well as being a current smoker, as independent risk factors for developing nosocomial sinusitis in this population.

Our findings demonstrate that 58.7% of general neurology ward patients with NGT intubation developed nosocomial sinusitis, compared to 67% of NCU patient with NGT intubation. This aligns with a previous study that observed a 67.9% paranasal sinus opacification rate in NCU patients with NGT intubation (9). Discrepancies exist when comparing to the 11.4% cumulative incidence of sinusitis from a sinus tap culture study in general ICU patients (7). Such disparities may reflect variations in clinical populations, diagnostic criteria, and tube sizes. Diagnosing sinusitis based on brain imaging criteria, rather than culture criteria, may result in an overestimation of its prevalence. In particular, the use of a larger bore tube (such as NGT), compared to smaller bore nasoenteric tubes likely used in the earlier study, could mechanically disrupt sinonasal microenvironment more severely. Our finding emphasizes the necessity of routine screening for nosocomial sinusitis for neurocritical patients, especially those with NGT intubation.

Our findings highlight the association between NGT and/or ETT intubation and the incidence of nosocomial sinusitis, demonstrating a dose–response relationship: patients with either NGT or ETT intubation exhibited a 2.60-fold increased risk, which escalated to a 6.17-fold increased risk with dual intubation. This pattern is consistent with previous analysis of the United States Nationwide Database (2008–2013), which showed 41 and 200% increased risks for ETT-only and dual intubation among acute hospital patients, respectively (17). Mechanistically, the insertion of foreign bodies may facilitate biofilm formation by serving as a scaffold for pathogen adhesion and impair mucociliary clearance through mechanical irritation (10). Additionally, NGTs may foster gastroesophageal reflux (18), enabling retrograde bacterial migration from the oropharynx to the paranasal sinuses. Our findings indicate the necessity of minimizing unnecessary intubation duration and considering alternative enteral access methods, such as postpyloric feeding through nasoduodenal or nasojejunal tubes, which reduce both tube caliber and reflux risk (19), and may consequently lower the incidence of nosocomial sinusitis.

We identified that current smokers had a 2.53-fold increased risk for developing nosocomial sinusitis among neurology patients with NGT intubation and NCU patients, and a 3.47-fold increased risk in NCU patients specifically. However, this risk did not generalize to former smokers, suggesting that smoking cessation may reduce this risk over time. Previous population-based studies suggest that smokers have a 44% higher risk of developing chronic rhinosinusitis than non-smokers (20), with current smokers facing a 12% higher risk than former smokers (21). This aligns with our findings, highlighting the reversibility of risk after quitting. Smoking may predispose individuals to chronic rhinosinusitis by impairing mucociliary clearance of the nasal mucosal epithelium, disrupting innate immune responses, and inducing sinonasal mucosal metaplasia (22). Although smoking did not directly induce nosocomial sinusitis, it might have contributed to alterations in the nasal mucosal environment. The combined effects of the intranasal tubes and smoking could accelerate changes in the nasal mucosal environment, and potentially facilitate biofilm formation (23). Smoking is also a well-established risk factor for cerebrovascular diseases (14). Therefore, we highlight smoking as a modifiable risk factor for nosocomial sinusitis and emphasize the importance of smoking cessation as a crucial tertiary prevention measure that could decrease both stroke recurrence and nosocomial sinusitis.

This study identified prolonged hospital stay as an independent risk factor for nosocomial sinusitis in the NCU population. Although these data cannot establish causality between extended hospital stays and nosocomial sinusitis incidence, the heightened prevalence of nosocomial infections within critical care environments (24) likely increases pathogen exposure and transmission among vulnerable NCU patients. This is particularly concerning for drug-resistant pathogens, which complicate treatment (16). Consequently, neurocritical patients who develop nosocomial sinusitis may face increased susceptibility to secondary infections, particularly pneumonia, and experience prolonged hospitalization. This aligns with previous observations linking nosocomial sinusitis to hospital-acquired pneumonia (13) and is supported by our finding of significantly higher pneumonia rates and longer hospital stays in patients with nosocomial sinusitis compared to those without. Critically, the potential pathway from nosocomial sinusitis to mechanical ventilation-associated pneumonia underscores the urgency for timely intervention, as it may precipitate life-threatening sequelae, including sepsis and death (3, 25). Future studies should explicitly compare the incidence of hospital-acquired pneumonia between NCU patients and general neurology patients with NGT intubation.

The primary strength of our study is that we included patients without sinusitis based on their initial head scan following admission. We also collected the date and location of NGT and ETT insertion, as well as the type of the tube used. Therefore, it is possible to determine cause-and-effect relationships between most of our variables in multivariate logistic regression. Moreover, we collected the smoking status of participants, which allows us to consider the impact of smoking status on the development of nosocomial sinusitis. Furthermore, as it is common to transfer neurocritical patients to general neurology wards following their NCU stay, to reduce the impact of NCU stay on general neurological patients with NGT intubation, patients who had NCU stays for two or more days were excluded from this study to minimize the potential impact.

### 4.1 Limitations

This study has several limitations. First, its single-center retrospective design inherently constrains the generalizability of findings. Second, the diagnosis of sinusitis relied solely on head imaging rather than nasal endoscopy or sinus puncture, primarily because the majority of patients presented a critical neurological condition with impaired consciousness. We also excluded patients with baseline sinusitis-positive imaging or those undergoing only one imaging session during hospitalization. Additionally, antibiotic treatment for concurrent nosocomial infections could have led to prophylactic antimicrobial effects prior to sinusitis development, potentially causing over- or underestimation of the reported nosocomial sinusitis incidence. Third, the onset of nosocomial sinusitis was defined by the first positive imaging after negative baseline imaging. The absence of routine sinusitis screening may overestimate the infection-free survival interval; consequently, survival analysis was not performed. Furthermore, non-concurrent enrollment of patients from NCU and general neurology wards introduces possible seasonal bias, though our analysis revealed no significant seasonal differences during hospital stays between patients with and without sinusitis. Future prospective studies with larger samples should incorporate more associate factors (e.g., the use of proton-pump inhibitor and immunosuppressants, and comorbidities) to validate and extend our findings.

### 4.2 Implications of nursing practice

An understanding of predisposing factors for nosocomial infections is critical for guiding clinical evaluations and formulating effective preventative strategies. Our findings have important implications for shaping intervention protocols that target the reduction in nosocomial sinusitis occurrence among neurology patients with NGT intubation and NCU patients. We recommend routine screening for nosocomial sinusitis by physicians and nursing staff for such patients. Furthermore, preventive measures such as promoting smoking cessation and exploring alternative feeding methods [e.g., antibiotic-coated catheters (16) or postpyloric feeding (19)] for long-term NGT intubation may lower the risk and consequential effects of nosocomial sinusitis. Furthermore, various clinical interventions, such as swallowing function rehabilitation for the removal of the NGT as early as possible and to reduce NCU stay by offering comprehensive treatment should be considered.

## 5 Conclusion

In this study, we identified NGT and/or ETT intubation, being a current smoker, and prolonged NCU stay as independent risk factors for the development of nosocomial sinusitis in patients with neurological conditions. These findings highlight the importance of early detection and management of nosocomial sinusitis, particularly in neurocritical patients with NGT intubation. We recommend that healthcare policymakers and professionals promote smoking cessation as proactive preventive measures to reduce the occurrence of nosocomial sinusitis. Further investigation into alternative methods and preventive strategies are crucial to reduce the incidence of this complication and, in turn, improving healthcare outcomes for individuals in neurocritical care environments.

## Data Availability

The datasets presented in this article are not readily available because of privacy or ethical restrictions but are available from the corresponding author upon reasonable request. Requests to access the datasets should be directed to liaoxy@gzhmu.edu.cn.
